# Reducing the urge to be physically active in patients with anorexia nervosa through virtual reality: protocol for a randomised-controlled feasibility trial

**DOI:** 10.1136/bmjopen-2024-097886

**Published:** 2025-01-30

**Authors:** Georg Halbeisen, Nina Timmesfeld, Georgios Paslakis

**Affiliations:** 1 University Clinic for Psychosomatic Medicine and Psychotherapy, Medical Faculty, Ruhr University Bochum, Bochum, Germany; 2 Department of Medical Informatics, Biometry & Epidemiology, Ruhr University Bochum, Bochum, Germany

**Keywords:** Eating disorders, Psychosocial Intervention, Virtual Reality

## Abstract

**Introduction:**

Weight restoration is a primary goal in anorexia nervosa (AN) treatment. Recent studies suggest that addressing physical activity urges in patients with AN is a promising target to facilitate weight restoration. This trial will evaluate the feasibility of a virtual reality (VR)-based intervention as an add-on treatment to psychotherapy to improve activity urges and, consequently, initial treatment responses on core outcomes as targeted per AN treatment guidelines.

**Methods and analysis:**

This single-centre feasibility trial adopts the single-blind, two-arm design and outcome measures of an intended full-scale randomised controlled trial (RCT) in order to establish that all necessary trial components work together as intended. It will evaluate feasibility as the primary endpoint and compare changes in ratings of the urge to be active between patients with AN randomly assigned to receiving VR intervention sessions and patients with AN in a control procedure. The feasibility of the full-scale RCT will depend on whether patients (1) will evaluate the experience as acceptable, (2) tolerate VR side effects and (3) will adhere to the intended intervention schedule. We define a set of three-tiered, formal progression criteria and employ a ‘traffic light system’ demarcating go (green), amend (amber) and stop (red) signals for advancing to the full-scale RCT.

**Ethics and dissemination:**

The study was approved by the ethics committee of the Ruhr University Bochum’s Medical Faculty at Campus East-Westphalia (AZ 2024-1296, 9 December 2024). Patients have to provide written consent before taking part in the study. The findings will be published with open access.

**Trial registration number:**

DRKS00035681, German Clinical Trials Register.

**STRENGTHS AND LIMITATIONS OF THIS STUDY**

- The feasibility trial adopts the single-blind, two-arm design and outcome measures of the intended full-scale randomised controlled trial.
- Sample size estimation is based on a formalised set of feasibility criteria.
- The study is monocentric.
- Objective physical activity levels are not assessed.

## Introduction

### Background and rationale

 Anorexia nervosa (AN) is a severe mental disorder that typically begins during adolescence and is characterised by significant weight loss or failure to gain weight appropriately for age due to restrictions of energy intake, an intense fear of weight gain and a disturbed body image.^1^ Its subgroups include a restricting type and a binge-eating/purging type, presenting with episodes of eating large amounts of food followed by weight control behaviours such as self-induced vomiting. AN exhibits the highest prevalence and incidence rates among young women and adolescent girls, with lifetime prevalence estimates ranging from 1.7 to 3.6%^2^ and yearly incidence ranging from 120.0 to 318.9 per 100 000 individuals.^3^ In men, prevalence and incidence rates are typically lower by a factor of 10 or more than in women,^4^ although rising steadily over the last three decades.^5^ Highlighting its substantial burden, patients with AN show increased health service utilisation^6^ and an all-cause mortality risk that is 5.9 times higher than in the general population, placing AN among the most lethal mental disorders.^7^ Estimates for Western Europe suggest that, in 2019, premature death and lost quality of life due to AN were responsible for 112.27 disability-adjusted life years per 100 000 individuals.^8^ The treatment and economic costs of AN average €319 million yearly.^9^

#### AN treatment targets

Weight restoration is a primary goal in AN treatment.^10^ Weight restoration is critical for medical stabilisation and sets the stage for long-term recovery.^11^ Studies identified weight gain across inpatient and outpatient settings as a positive predictor of recovery from AN up to 1 year after treatment,^12^,^15^ with early weight gain during the first 3–4 weeks of treatment being a significant predictor of clinical outcomes.^16^,^19^ Next to refeeding and nutritional rehabilitation, psychotherapy for adults and family-based treatments for adolescents are considered first-line treatment options.^20^,^22^ Although treatment often results in temporary weight restoration, patients with AN remain at an exceedingly high risk for relapse, with the majority of studies reporting relapse rates greater than 25% and up to 52% within the first year after successful treatment.^23^ The volatility of treatment responses highlights that addressing maintaining factors of low body weight in AN remains a major challenge^24^ and that novel treatment approaches to augment existing options are urgently needed.^25^

#### Unaddressed maintaining factors of low body weight

Recent studies suggest that addressing physical activity (PA) urges in patients with AN is a promising target to facilitate weight restoration.^26^,^29^ Up to 75% of patients with AN, irrespective of the subtype, show increased PA levels^30^ despite experiencing tiredness and fatigue.^31^,^33^ PA, broadly referring to any body movement produced by skeletal muscle contractions as part of both exercising and non-exercising (eg, standing, walking or fidgeting), substantially increases energy expenditure relative to basal metabolism^34 35^ and contributes to the maintenance of low body weight.^36^,^39^ In a systematic review of the literature, Rizk *et al*^30^ proposed that the urge to engage in PA in patients with AN develops gradually. Initially, individuals engage voluntarily in PA to maximise weight loss at early stages of AN development. However, with increasing AN severity and PA intensity, engaging in PA transitions into a coping strategy to alleviate negative affect and body image concerns, ultimately evolving into an autonomous and compulsive activity drive characterised by diffuse restlessness and aimless, involuntary PA.^33 40^

Increased levels of PA have been linked to the severity of AN psychopathology and associated to longer hospitalisations, poorer treatment outcomes (shorter times to relapse, chronic course of the disorder) and lower life quality.^2931 37 41^,^43^ To facilitate weight gain during nutritional rehabilitation (especially in the initial stages of treatment and for patients with very low weight), abstinence from PA is recommended.^44^ However, patients with AN experience prescribed inactivity as detrimental to their mental well-being and to the management of their condition.^45^ Patients may also still engage in non-exercising PA, such as deliberate (eg, isometric) muscle contractions or more automatic fidgeting, which are difficult to monitor and can negatively impact weight restoration.^46^ Thus, there is a continuing and unmet need to address the urge to be physically active during AN treatment.

#### The potential of virtual reality (VR)-based interventions

In the absence of interventions targeting activity urges, we developed and piloted a concept for a novel VR-based intervention.^28^ Based on the efficacy of VR body exposure techniques,^47^,^53^ we hypothesised that simulated movement within a highly immersive 360° VR environment would support the illusion of actual body movements and effectively alleviate patients’ urge to engage in PA. In a single-arm, proof-of-concept study,^28^ 20 patients with AN, who were receiving inpatient therapy, were exposed to 30 min of simulated PA (ie, jogging) from first-person perspective. A 360° camera had been used to professionally create a three-dimensional jogging sequence in a countryside scene. Patients wore VR goggles and followed a running track from a first-person perspective, simulating the experience of jogging themselves. Patients were able to turn their head to all sides while jogging, providing sensory information critical to creating a sense of immersion.^54^ Before and after the session, patients were asked to rate their acute urge to be physically active using a self-report questionnaire that captured cognitive, emotional and behavioural aspects.^55^ Pointing towards the utility of simulated VR movement, the single session of VR jogging led to a 14% reduction in acute activity urge scores from baseline to post session, with the within-session habituation onset (ie, reduction from peak activation) occurring after 12 min. Associations between increased autonomic nervous activity (α-amylase in saliva) and the observed reductions in the urge to move suggested a critical role of physiological activation due to the virtual jogging in explaining the effect. These findings suggest initial proof-of-concept and substantiate the main hypothesis that engaging in virtual movement decreases activity urges in patients with AN.

### Objectives

Based on recommendations for the phase-based development of novel interventions,^56^ this feasibility trial aims to obtain information required to design a full-scale, multicentric, single-blind, two-arm randomised controlled trial (RCT). The full-scale RCT will examine the effectiveness of the VR-based intervention as an add-on treatment to psychotherapy to improve activity urges and, consequently, initial treatment responses on core outcomes as targeted per AN treatment guidelines (ie, weight gain, reduction in eating disorder psychopathology). However, to advance to the full-scale RCT, it is essential to first determine feasibility. Patients may find using the VR intervention unacceptable, too difficult or cumbersome, or they may not tolerate VR-induced side effects (such as ‘cyber sickness’, see below). This could result in premature termination of trial sessions, which would threaten the hypothesised exposure effects that require minimising avoidance behaviour,^57^ and thus jeopardise completing exposure sessions. Our primary objective is to determine whether patients (1) evaluate the intervention as acceptable, (2) tolerate the interventions’ side effects and (3) will adhere to the intended intervention schedule. Our secondary objective is to estimate and assess the between-group effect of the intervention on reducing activity urges as well as the SD of the full-scale RCT’s outcome parameters, to determine the desired treatment effect and calculate the full-scale RCT’s required sample size.

### Trial design

**Figure 1:**
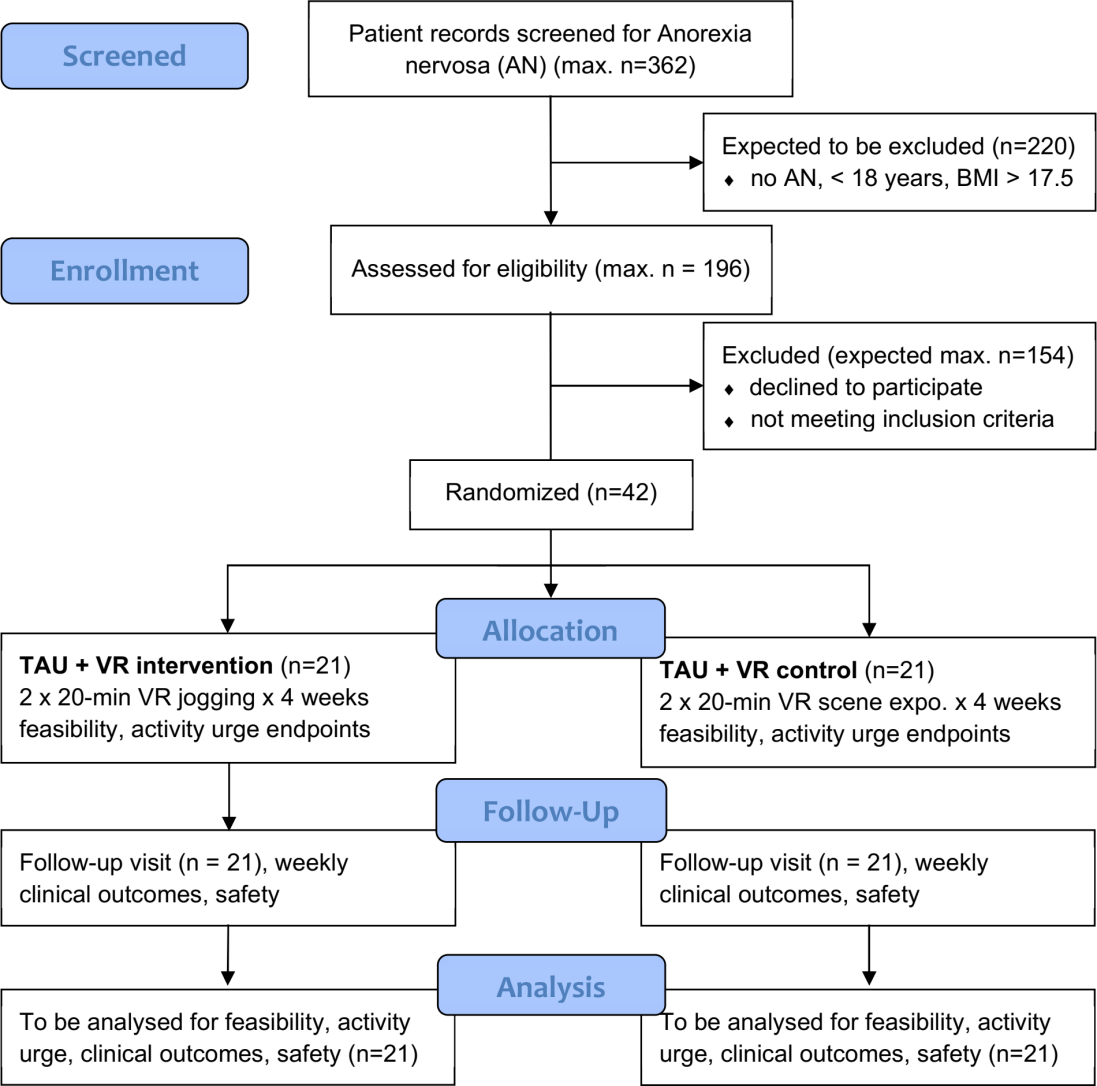
Trial design visual depiction. BMI, body mass index; TAU, treatment as usual; VR, virtual reality.

The single-centre feasibility trial adopts the single-blind, two-arm design and outcome measures of the intended full-scale RCT in order to establish that all necessary trial components (eg, randomisation, blinding, the control intervention) work together as intended. It will evaluate feasibility as the primary endpoint and compare changes in ratings of the urge to be active between patients with AN randomly assigned to receiving VR jogging sessions and patients with AN in a control procedure (see figure 1). Progression towards the full-scale RCT will depend on meeting a set of three-tiered formal progression criteria of a ‘traffic light system’ with go (green, ie, acceptable feasibility), amend (amber, ie, potential feasibility pending trial modifications) and stop (red, ie, unacceptable feasibility) continuation signals.^58^ We will not progress to the full-scale RCT if at least one stop signal is obtained.

## Methods and analysis

### Study setting

Trial participants with AN will be recruited over the course of 16 months from one of the largest specialised eating disorder treatment facilities in Germany, located in a rural area in North Rhine-Westphalia, among inpatients receiving a multimodal disordered eating treatment based on psychodynamic and cognitive–behavioural approaches (ie, treatment as usual (TAU)). The facility’s admission statistics show that an average of 362 patients present with an eating disorder throughout a year, with 196 patients treated in 2022 meeting the key inclusion criteria for the trial (see below).

### Eligibility criteria

The trial will include patients admitted to inpatient treatment with a diagnosis of restricting or binge-eating/purging type AN according to International Classification of Diseases, Tenth Revision (ICD-10) criteria (ie, F50.00 and F50.01), with a body mass index (BMI)≤17.5 kg/m², aged 18 years or older, able and willing to provide informed consent. Only adult patients with AN will be included to reduce the influence of maturation and increase the homogeneity of treatment schedules and procedures (ie, adolescents receive family-based treatment). Patients will be recruited within the first week after admission to inpatient treatment, as we expect activity urges to be most pronounced during this phase due to the start of nutritional rehabilitation.

We will exclude patients with AN who present with severe comorbid psychiatric or somatic disorders or other conditions that may interfere with study protocol adherence and/or ability to provide informed consent (eg, severe depression, acute suicidal tendencies, relevant difficulties in spoken and written German). We explicitly refrain from excluding patients based on sex to increase sample diversity,^59^ which will benefit the generalisability of findings.^60^ However, we expect to include a significantly higher number of women due to skewed base rates in the patient population, which we will account for in statistical analyses.

### Interventions

Eligible patients will be randomised to receive the VR intervention or a VR control procedure in addition to TAU, which includes individual and group psychotherapy, psychoeducation, nutritional rehabilitation and complementary therapies (eg, art therapy). Patients will be blinded as per the different trial conditions, and we will encourage patients not to share their specific VR experiences with other therapists or patients. However, to avoid expectancy biases, all patients will be informed that the procedure aims to reduce activity urges. Both intervention and control procedures comprise 8×30 min sessions (20 min of exposure +10 min of preparation and follow-up). All sessions will be delivered by a trained doctoral researcher, with the principal investigator providing clinical supervision.

During the intervention, patients with AN will be exposed to a 20 min simulated experience of a 3D jogging sequence in a countryside scene. We chose jogging as a PA which most people may easily relate to. While standing, patients will be wearing a head-mounted display and watch a 360° video of a jogger’s running track from the first-person perspective, as if it were them actually jogging. Our previously described published pilot study showed that an average within-session habituation onset (ie, reduction from peak activation) can be expected to occur after 12 min of exposure,^28^ suggesting that 20 min sessions should produce sufficient levels of within-session effects.^57^

Following recommendations for trials with non-pharmacological interventions,^61^ we will compare the novel VR intervention to a control intervention to account for unspecific intervention components, including the amount of attention and empathy, and patient outcome expectations. Specifically, patients with AN randomly assigned to the control procedure will be exposed to a 3D, 360° stationary VR environment (similar to scenes from the jogging sequence) that have been pre-rated in terms of valence and arousal^62^ for a total duration of 20 min. They will be able to turn their head and look around but will not be exposed to any vicarious movement, and will thus be deprived of the intervention’s active component.

### Outcomes

Primary endpoints include established measures of VR intervention feasibility^63^: acceptability will be measured with the System Usability Scale (SUS)^64^; tolerability will be measured with the Simulation Sickness Questionnaire (SSQ)^64 65^; intervention adherence is measured in minutes of completed VR exposure. We will administer all assessments on a per-session basis and use the means across sessions as dependent variables. Acceptable levels of feasibility (ie, the trial progression criteria) for the primary endpoints are defined as follows: (1) normative data for the SUS suggest that average scores below 50 indicate insufficient acceptability, and scores above 70 indicate sufficient acceptability^66^; (2) empirical evidence shows that 60–95% of participants experience some level of cyber sickness during VR exposure, as determined by an SSQ Score greater than 40.^67 68^ Given its high prevalence, we deemed that cyber sickness rates (ie, proportion of patients with an average, across-session SSQ scores≥40) exceeding 95% would clearly indicate the RCT as infeasible, whereas any rates below 60% would suggest the RCT is feasible; (3) adherence, according to our pilot data, refers to completing at least 12 min of exposure, at which point habituation onset can be observed. We reasoned that adherence rates should not be lower than 50% (ie, no fewer than half of the patients should complete the minimum amount of average exposure) and that it should ideally approach the average level estimated across other VR exposure therapies (84%^69^).

Secondary endpoints include (1) outcomes relevant to the mechanism of the intervention (ie, activity urges) and (2) clinical outcomes (to be evaluated in the full-scale RCT) as targeted by AN treatment guidelines.^21^ Activity urges will be recorded on a per-session basis using the validated state activity urges questionnaire (State Urge to be Physically Active-Questionnaire (SUPA-Q)).^55^ The clinical outcomes, monitored on a weekly basis, include changes in eating disorder psychopathology using the Eating Disorder Examination-Questionnaire (EDE-Q),^70^ as well as weekly weight gain, defined by changes in patient BMI (in kg/m²), which will be obtained from patient records.

Safety and additional assessments include the rate of weight gain and potential moderators of intervention effects. Weight restoration is one of the primary goals in AN treatment.^10^ Current treatment guidelines propose weight gains of 500 g per week,^21^ though this is rarely achieved in a steady manner unless in tightly controlled settings.^24^ We reasoned the intervention would be unsafe, if successful weight gain rates in the intervention condition fall below the weight gain rates in the control condition. Potential moderators of intervention effects, to be evaluated in the full-scale RCT, include the Commitment to Exercise Scale (CES),^71^ and excessive PA status in the 28 days prior to admission, which we will assess at trial start during a face-to-face interview and categorise using the criteria: (1) ≥1 hour a day and ≥5 times a week, (2) vigorous intensity level, (3) exercise for weight or shape reasons and (4) perceived ‘obligatory’ character. Therapy motivation will be examined using the therapy motivation questionnaire (Fragebogen zur Messung der Psychotherapiemotivation (FPTM-23)).^72^ We will further conduct semistructured trial completion interviews. This includes a ‘funnelled debriefing’ procedure (ie, asking increasingly specific questions about the trial hypothesis and different treatment arms) to evaluate patient blinding,^73^ and open-ended questions concerning reasons for trial dropout or premature cessation, if applicable.

### Participant timeline

Because changes during the first 3–4 weeks are critical for overall AN treatment outcomes,^16^,^18^ the VR intervention or control procedure will be delivered on a weekly basis over the course of the initial four consecutive weeks of AN treatment. On admission, we will screen patient records based on diagnosis, age and BMI. Patients potentially meeting inclusion criteria will be assessed for eligibility and, if eligible and willing to participate, randomised to receive the VR intervention or VR control procedure on a weekly basis over the course of the four consecutive weeks. A weekly schedule with two sessions will be implemented as we expect daily experiences (eg, supervised meals, weighing and mirror exposure as part of regular inpatient treatment) to trigger activity urges repeatedly, thus requiring regular and repeated attendance. The proposed trial duration and frequency of visits are similar to other VR-based exposure therapies for patients with AN.^49 52 74^

**Figure 2:**
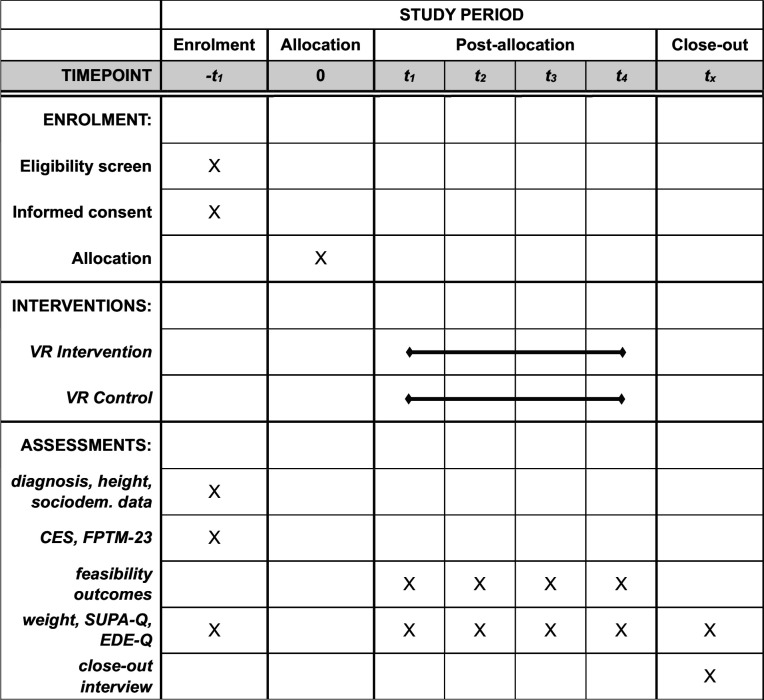
Participant timeline schematic diagram. CES, Commitment to Exercise Scale; EDE-Q, Eating Disorder Examination-Questionnaire; FPTM-23, Fragebogen zur Messung der Psychotherapiemotivation; SUPA-Q, State Urge to be Physically Active-Questionnaire; VR, virtual reality.

At enrolment, we will record patient height and body weight, and diagnosis (based on ICD-10 criteria), and patients will be asked to complete a sociodemographic questionnaire, the EDE-Q, the CES, the FPTM-23 and a brief excessive PA interview. At the end of each week during the trial, patients will complete the SUPA-Q, the EDE-Q, and we will record patients’ current weight. After each session, patients will provide acceptability and tolerability ratings, and we will record the duration of completed exposure sessions. We will record the reasons, if sessions are terminated prematurely. At close-out, we will evaluate patient blinding in a close-out interview, and record patient weight, SUPA-Q and EDE-Q scores (see figure 2).

### Sample size

We employed a hypothesis testing approach for feasibility outcomes developed by Lewis *et al*^75^ to determine the sample size for the proposed feasibility trial. The rationale of this approach is that we determine a sample size that will allow us to test an observed feasibility outcome (eg, the adherence rate) against being in the defined red zone (unacceptable rate), based on an expectation of it being in the defined green zone (acceptable rate). In other words, we define a null hypothesis (the true feasibility outcome is not greater than the value demarcating the red zone) and an alternative hypothesis (the true feasibility outcome is greater than the value demarcating the red zone by magnitude of its difference towards the value demarcating the green zone). The study is then powered for a one-sided test to detect the difference between red and green zones. The amber zone (between red and green) is then split into two areas (major and minor amend) by the critical boundary of the corresponding test. For the proposed trial, α (one tailed) and β were set to.05.

The sample size calculations are listed in table 1 and were based on binomial exact tests (for tolerability and adherence rates) and a one-sample t-test (for acceptability means), respectively. The maximum sample size suggested by the Lewis approach is n=21 for evaluating adherence. We therefore targeted the recruitment of 42 patients to be randomly allocated to either the intervention arm or the control condition for an intention-to-treat analysis.

**Table 1:** Sample size calculations

Feasibility outcome	Green zone	Red zone	Required n*
Acceptability (mean SUS across patients; SD=22.5^84^)	>70	<50	16
Tolerability (% of patients with SSQ mean≥40)	<60	>95	14
Adherence (% of patients with exposure mean≥12 min)	>84	<50	21

Note. Observed values in-between green and red zones provide no clear evidence for or against feasibility. This may signal to discuss steps to amend the trial procedures or re-evaluate progression criteria. *n designates the minimum number of recruited per study arm based on binomial exact tests (for Tolerability and Adherence rates) and a one-sample -test (for Acceptability means), respectively.

* n designates the minimum number of recruited patients per study arm based on binomial exact tests (for tolerability and adherence rates) and a one-sample t-test (for acceptability means), respectively.

SSQSimulation Sickness QuestionnaireSUSSystem Usability Scale

For the analysis, the ‘traffic light’ approach will be applied. Each outcome is considered separately, and overall progression is then determined by the worst performing criterion (ie, go, if all outcomes are green; stop, if one outcome is red; amend, if one outcome is in the amber zone, but none are red). The probabilities for the wrong decision for the three feasibility outcomes with the planned sample size of 21 per arm is shown in table 2. Combined, the probability of falsely not progressing to the full trial is smaller than the sum of probabilities to falsely fall within the red zone and hence is smaller than 0.07%.

**Table 2:** Probabilities for the wrong decision for the three feasibility outcomes

Probability if true value within	Red zone and falsely observed value within green zone	Green zone and falsely observed value within red zone
Acceptability	0.03%	0.03%
Tolerability	<0.01%	0.03%
Adherence	0.07%	0.01%

### Recruitment

We will screen patients and assess patients for eligibility until achieving the target sample size. Admission statistics of the study centre reveal that an average of 362 patients present with an eating disorder (ED) throughout a year, with 196 patients treated in 2022 meeting the key inclusion criteria for the trial (adult, AN diagnosis, BMI<17.5 kg/m²). Based on these numbers and the analysis goal, we targeted the recruitment of 42 patients in a 16-month window. This corresponds to a recruitment rate of approximately 22% (the equivalent of two to three patients per month).

### Assignment of interventions

Participants will be randomly assigned to either control or intervention conditions with a 1:1 allocation as per a specified randomisation schedule using permuted blocks of random sizes. Randomisation will be conducted by a staff member not involved in the recruitment or interviews. An independent researcher blinded to the patient’s intervention group will conduct all interviews and transfer the data into the database to avoid any detection bias. Blinding of the researcher administering the intervention was deemed infeasible, but patients will be blinded as per the different trial conditions, and we will encourage patients to not share their specific VR experiences with other therapists or patients. The researcher administering the intervention will otherwise not be involved in the patient’s overall treatment, such that performance biases are unlikely.

### Data collection methods

All quantitative data will be collected pseudonymously via electronic data capture tools (see below). ICD-10-based diagnosis, height and medications will be obtained from patient records. Patients will self‐report their age, sex, sexual orientation, German language ability, migration background, years of education, marital status, living circumstances, days in current treatment and previous treatments. Acceptability will be measured with the SUS.^64^ The SUS is a widely used instrument that measures the subjective usability of products and systems with 10 questions on a 5-point scale (from *strongly disagree* to *strongly agree*), which, after linear transformations, are scored on a range from 0 (low usability) to 100 (high usability). The German SUS^76^ has good reliability (Cronbach’s α=0.84). Tolerability will be measured using the SSQ.^64 65^ The SSQ asks patients to rate 16 symptoms on a scale from 0 to 3 (none, slight, moderate and severe), with a weighted sum score (Cronbach’s α=0.84) indicating overall severity.^77^ Intervention adherence is measured in minutes of completed VR exposure using a stop watch.

Patient weight will be assessed using a calibrated scale (KERN & SOHN GmbH, Balingen-Frommern, Germany). Activity urges will be recorded using the validated state activity urges questionnaire (SUPA-Q).^55^ Its 21 items assess current activity urges (eg, ‘I have the urge to be physically active right now’) on a 5-point scale from 1 (not at all, 0%) to 5 (a great deal, 100%) and can be aggregated into a total score reflecting a single latent factor (Cronbach’s α=0.95). Eating disorder psychopathology is assessed with the EDE-Q.^70^ The EDE-Q contains 22 attitudinal items that can be combined into a global score of cognitive and behavioural ED symptoms over the last 28 days (Cronbach’s α=0.94) rated on a 7-point scale (from 0, never, to 6, every day). We will use a version with a slightly modified instruction to assess ED symptoms within the past week. The CES,^71^ with eight items rated on a visual analogue scale (horizontal line of 12.2 cm) with bipolar adjectives on each end, will be used to assess obsessive–compulsive aspects of PA (Cronbach’s α=0.82). Excessive PA status in the 28 days prior to admission will be assessed during a face-to-face interview and categorised using the criteria: (1) ≥1 hour a day and/or ≥5 times a week, (2) vigorous intensity level, (3) exercise for weight or shape reasons and (4) perceived ‘obligatory’ character. Therapy motivation will be examined using the therapy motivation questionnaire (FPTM-23).^72^ The FPTM-23 includes 23 items rated on a 4-point scale (agree, somewhat agree, somewhat disagree, disagree), which are summed into various subscales (Cronbach’s α≥0.78).

### Data management

Data management will be done using Research Electronic Data Capture (REDCap) electronic data capture tools. REDCap^78 79^ is a secure, web-based software platform designed to support data capture for research studies, providing (1) an intuitive interface for validated data capture; (2) audit trails for tracking data manipulation and export procedures; (3) automated export procedures for seamless data downloads to common statistical packages; and (4) procedures for data integration and interoperability with external sources.

### Data analysis

For all primary outcomes, rate and corresponding 95% CI will be estimated with the method of Clopper-Pearson. Furthermore, for each estimated rate, it will be checked whether it is inside the red, amber or green zone. If at least one rate falls within the red zone, a decision against feasibility is made.

Repeated measures of the SUPA-Q Score will be analysed with a linear mixed effect model, using the score after the intervention as dependent variable, the score from the same day before intervention and the intervention group as fixed effect, and a random patient effect. For the other secondary outcomes, weight gain and EDE-Q, exploratory analysis will be done with a linear model (analysis of covariance (ANCOVA)), with the value at the end of treatment as dependent variable and the intervention group, the baseline value and AN subtype as independent variables.

All analyses will be done in the intention-to-treat population. For all feasibility outcomes, missing values will be imputed taking the reasons for dropout into account. We will not conduct interim analyses.

### Data monitoring

Following International Conference on Harmonisation - Good Clinical Practice (ICH GCP) guidelines for non-drug interventional trials outside the jurisdiction of German Pharmaceutical Law,^80^ we devised a monitoring and data reviewing strategy according to estimated risks and needs of the current trial. To ensure protocol adherence and compliance with local data protection policies and reporting obligations, involved staff will be trained during initiation visits. All study documents will be handed over in person. Monitoring visits conducted three times per site will verify adherence to inclusion and exclusion criteria, ensure that informed consent was obtained, check that adverse events were recorded and reported and monitor recorded data for readability and completeness. Data quality and completeness will be checked again at close-out visits. All trial monitoring will be conducted independently by the Clinical Trials Centre Cologne.

Three renowned experts in the eating disorders field will serve as members of the trial’s international Data and Safety Monitoring Board (DSMB). The DSMB will be informed of all safety-related events and will monitor the safety and progress of the clinical trial in regular meetings.

### Patient and public involvement

First-hand experiences of patients with AN who reported the significant urge to constantly engage in PA directly informed the rationale of the present trial.^27 28^ Impairments in the ability to adhere to recommended therapeutic regimes and the persistence of activity urges despite behavioural modification attempts and supervised activities suggested a pressing need to develop additional therapeutic approaches as add-ons to existing psychotherapy. During the feasibility trial, we will establish a patient advisory board of three to four persons with current or previous AN. The patient advisory board will be consulted prior to recruitment to discuss and provide input on the study set-up, and after data collection to discuss and vote on the wording of a series of statements relating to clinical implications of findings. These will be included in the published results. The patient advisory board will also be consulted to discuss unexpected compliance and adherence issues. Meetings will be conducted online and anonymously.

## Ethics and dissemination

### Research ethics approval

The study is based on the guidelines of Good Scientific Practice of the German Research Foundation and was approved by the ethics committee of the Ruhr University Bochum’s Medical Faculty at Campus East-Westphalia (AZ 2024-1296, 9 December 2024).

### Consent

Participants are informed and educated about the intervention and study goals prior to data collection (for consent form, see online supplemental appendix); informed consent, as defined by the Declaration of Helsinki, is a condition for study participation.

### Confidentiality and dissemination policy

All data relevant to the trial is pseudonymised, digitally transferred to the statistical software and stored on a password-protected server that is only accessible to authorised employees. The use of the VR hardware and software does not require the collection of personal data from participants and these data are not collected or stored within the hardware or software. Data will only be transferred in pseudonymised form. Pseudonymised data will be stored separately from consent forms. All data collected as part of this study will be deleted after 10 years. Participants will also be informed of their rights under the European General Data Protection Regulation (as of 25 May 2018) and the Data Protection Act of North Rhine-Westphalia (as of 9 March 2021).

Trial reporting will adhere to the Consolidated Standards of Reporting Trials 2010 statement extension for randomised pilot and feasibility trials ^81^ and to Template for Intervention Description and Replication guidelines for reporting interventions.^82^ Data coding sheets, coded data, codebooks, analysis syntax, as well as any other non-confidential documents created as part of the trial will be made publicly available using the Open Science Framework (https://osf.io/) repository, adhering to Findable, Accessible, Interoperable, Reusable (FAIR) principles.^83^ The findings will be published with open access within 1 year after project conclusion.

### Protocol amendments

Any deviations from the original trial design will be documented in both the trial registry and the journal in which the study or studies will be published.

## supplementary material

10.1136/bmjopen-2024-097886 online supplemental file 1
