# Supplementary material for: Reducing the urge to be physically active in patients with anorexia nervosa through virtual reality: protocol for a randomised-controlled feasibility trial
Source: BMJ Open. 2025 Jan 30;15(1):e097886. doi: 10.1136/bmjopen-2024-097886 (PMC11784207; doi:10.1136/bmjopen-2024-097886)
Supplement: online supplemental file 1 [file bmjopen-15-1-s001.pdf]

Medizinisches Zentrum für Seelische Gesundheit | Virchowstraße 65 | 32312 Lübbecke

Universitätsklinik für Psychosomatische Medizin und Psychotherapie – Campus OWL

Univ.-Prof. Dr. med. G. Paslakis, MBA
Leitender Arzt

Virchowstraße 65
32312 Lübbecke

Tel. 05741 / 35-0 Zentrale
Tel. 05741 / 35-47-4500 Sekretariat
Tel. 05741 / 35-4011 Psychiatrische Ambulanz
Fax 05741 / 35-2710
E-Mail-Adresse: psychosomatik@muehlenkreiskliniken.de
www.muehlenkreiskliniken.de

Reduktion von Bewegungsdrang bei Anorexia Nervosa mittels virtueller Realität: Eine randomisiert-kontrollierte Machbarkeitsstudie

Patienteninformation und Einwilligungserklärung

Patienteninformation

Die Richtlinien guter klinischer Praxis (GCP) sehen vor, dass sich die Teilnehmer*innen an empirischen Studien explizit und nachvollziehbar einverstanden erklären, dass sie freiwillig an unserer Forschung teilnehmen. Aus diesem Grund möchten wir Sie bitten, die nachfolgenden Erläuterungen zum Inhalt der Studie zu lesen und anzugeben, ob Sie sich mit der Teilnahme an der Studie einverstanden erklären und der Verarbeitung Ihrer Daten zustimmen.

Prüfarzt und Prüfstelle dieser Studie

Univ.-Prof. Dr. med. Georgios Paslakis, MBA
Universitätsklinik für Psychosomatische Medizin und Psychotherapie
Virchowstraße 65 • 32312 Lübbecke
Telefon: 05741 / 35 – 4500 (Sekretariat) • Telefax: 05741 / 35 – 2710
E-Mail-Adresse: psychosomatik@muehlenkreiskliniken.de

Gegenstand der Studie

Personen mit Anorexia Nervosa („Magersucht") verspüren oft den Drang, übermäßig stark Sport zu treiben oder auf andere Weise körperlich aktiv zu sein, was die Gesundheit von Betroffenen negativ beeinflussen kann. Im Rahmen der Behandlung von Essstörungen wird der akute Drang nach körperlicher Aktivität jedoch selten berücksichtigt. Ziel der vorliegenden Studie, **„Reduktion von Bewegungsdrang bei Anorexia Nervosa mittels virtueller Realität: Eine randomisiert-kontrollierte Machbarkeitsstudie",** ist es, eine durch Virtuelle Realität (VR) gestützten Ansatz zu erproben, der den Drang nach übermäßiger Aktivität lindern soll. Virtuelle Realität (VR) beschreibt hierbei eine künstliche, am Computer erzeugte und über eine spezielle Brille dargestellte Umgebung, in der man sich scheinbar bewegen und Handlungen ausführen kann.

Ablauf der Studie

Diese Studie beinhaltet die Bearbeitung von Fragen zu Ihrer Person, Fragebögen Essstörungssymptomen und zu Bewegungsdrang sowie die wiederholte Teilnahme an und Bewertung einer Übung in „Virtueller Realität“, welche über eine Brille dargestellt wird.

Dauer

Die Studienteilnahme erstreckt sich über insgesamt 4 Wochen. Zum Studieneinschluss werden wir Sie bitten, eine Reihe von Fragebögen auszufüllen. Dies wird ca. 45 min in Anspruch nehmen. Anschließend werden wir mit Ihnen zwei Sitzungen pro Woche, für insgesamt 4 Wochen, von je 30 min vereinbaren. Die Sitzungen beinhalten je 20 min, in denen Sie die VR-Brille tragen und sitzend eine Übung bearbeiten, und 10 min, in denen Sie Fragen zur Übung und/oder weitere Fragebögen zum Bewegungsdrang und Essstörungssymptomen beantworten.

Möglicher Nutzen der Studie

Die Intervention dieser Studie hat zum Ziel, einen erlebten Drang nach Bewegung zu reduzieren. Es ist jedoch möglich, dass Sie aus Ihrer Teilnahme keinen direkten gesundheitlichen oder persönlichen Nutzen ziehen werden; künftige Patient*innen mit Essstörungen werden jedoch von den Ergebnissen dieser Studie profitieren.

Mit der Teilnahme verbundene Erfahrungen/Risiken

Die Teilnehmer*innen an dieser Studie werden keinem gesundheitlichen Risiko ausgesetzt, das über die Risiken des alltäglichen Lebens hinausgeht. Manche Personen erleben Übelkeit, wenn sie Bewegung in virtueller Realität sehen, ohne sich selber zu bewegen. Es steht Ihnen jederzeit frei, die Teilnahme zu beenden.

Erklärung zum Datenschutz

Die Datenverarbeitung dieser Studie geschieht nach datenschutzrechtlichen Bestimmungen der Datenschutz-grundverordnung (DSGVO) sowie des Datenschutzgesetzes Nordrhein-Westfalen (DSG NRW). Die Daten werden ausschließlich für die im Aufklärungsbogen beschriebenen Zwecke verwendet.

Im Rahmen dieser Studie werden folgende Daten erhoben:

- Fragebogen zur Erfassung von Essstörungssymptomen (EDE-Q)
- Fragebogen zur Erfassung von Bewegungsdrang (SUPA-Q)
- Fragebogen zur Erfassung von Simulationsübelkeit (SSQ)
- Fragebogen zur Erfassung der Simulationsbedienbarkeit (SUS)
- Fragebogen zur Erfassung der Psychotherapiemotivation (FPTM-23)
- Fragebogen zur Erfassung getriebenen Sporttreibens (CES)

Als personenbezogene Daten werden erhoben:

- Alter
- Geschlecht
- Deutschkenntnisse
- Bildungsstatus
- Körpergröße
- Körpergewicht (aus der Patientenakte)
- Medikation
- Zeit seit Erstdiagnose einer Essstörung
- Anzahl früherer Behandlungen
- Diagnose (aus der Patientenakte)

Vertraulichkeit

Alle im Rahmen dieser Studie erhobenen Daten sind selbstverständlich vertraulich und werden nur in pseudonymisierter Form genutzt. Demographische Angaben wie Alter oder Geschlecht lassen keinen eindeutigen Schluss auf Ihre Person zu.

Hinweis zur Nutzung von Produkten der Firma *Meta Platforms, Inc.*

In dieser Studie werden Produkte der Meta Platforms, Inc. verwendet (VR-Brille und Bedienteile, Software zur grafischen Darstellung). Die Verwendung dieser Produkte setzt keine Erfassung Ihrer personenbezogenen Daten voraus und diese Daten werden auch nicht innerhalb der Hardware- und Software erfasst oder gespeichert. Meta Platforms, Inc. erhält keinerlei Zugriff auf die hier erfassten Daten. Mit der Teilnahme erklären Sie sich jedoch grundsätzlich mit den Nutzungsbedingungen der Meta Platforms, Inc. einverstanden und erkennen an, dass sich durch die Verwendung der Produkte keinerlei Garantie-, Gewährleistungs- oder Haftungsansprüche gegenüber Meta Platforms, Inc. ergeben.

Aufbewahrung

Die mit dieser Studie erhobenen Daten werden am Standort der Universitätsklinik für Psychosomatische Medizin und Psychotherapie in Lübbecke gespeichert und nach 10 Jahren gelöscht. Die Speicherung erfolgt in einer Form, die keinen direkten Rückschluss auf Ihre Person zulässt, das heißt die Daten werden pseudonymisiert.

Freiwilligkeit & Rechte der Versuchspersonen

Ihre Teilnahme an dieser Untersuchung ist freiwillig. Es steht Ihnen bis zur Veröffentlichung der Ergebnisse der Studie frei, Ihre Teilnahme abzubrechen und damit diese Einwilligung zurückziehen (Widerruf), ohne dass Ihnen daraus Nachteile entstehen. Wenn Sie die Teilnahme abbrechen, werden wir Sie fragen, ob wir die bis zum Zeitpunkt des Widerrufs erhobenen Daten verwenden dürfen; sollten Sie dem nicht zustimmen, dann werden alle vorliegenden Daten zu Ihrer Person vernichtet.

Sie haben das Recht, Auskunft über die Sie betreffenden personenbezogenen Daten zu erhalten sowie ggf. deren Berichtigung oder Löschung zu verlangen. In Streitfällen haben Sie das Recht, sich beim Datenschutzbeauftragten der Universitätsklinik für Psychosomatische Medizin und Psychotherapie Lübbecke oder bei den Datenschutzbeauftragten des Landes NRW zu beschweren (Adresse siehe Einverständniserklärung).

Universitätsklinik für Psychosomatische Medizin und Psychotherapie
Campus OWL
Univ.-Prof. Dr. med. G. Paslakis, MBA
Leitender Arzt
Virchowstraße 65 • 32312 Lübbecke
Telefon: 05741 / 35 – 4500 (Sekretariat) • Telefax: 05741 / 35 – 2710
psychosomatik@muehlenkreiskliniken.de • www.muehlenkreiskliniken.de/psychosomatik

Klinik am Korso gGmbH
[personal information redacted]

Medizinisches Zentrum für Seelische Gesundheit | Virchowstraße 65 | 32312 Lübbecke

Universitätsklinik für Psychosomatische Medizin und Psychotherapie – Campus OWL

Univ.-Prof. Dr. med. G. Paslakis, MBA
Leitender Arzt

Virchostraße 65
32312 Lübbecke

Tel. 05741 / 35-0 Zentrale
Tel. 05741 / 35-47-4500 Sekretariat
Tel. 05741 / 35-4011 Psychiatrische Ambulanz
Fax 05741 / 35-2710
E-Mail-Adresse: psychosomatik@muehlenkreiskliniken.de
www.muehlenkreiskliniken.de/psychosomatik

Reduktion von Bewegungsdrang bei Anorexia Nervosa mittels virtueller Realität: Eine randomisiert-kontrollierte Machbarkeitsstudie

Einwilligungserklärung

Ich habe die Erläuterungen gelesen und bin damit einverstanden, an der oben genannten Studie teilzunehmen. Ich hatte Gelegenheit, Fragen zu stellen und Unklarheiten zu beseitigen. Ich habe eine Kopie dieser Erklärung erhalten.

Ich erkläre mich einverstanden, dass die im Rahmen der Studie erhobenen Daten zu wissenschaftlichen Zwecken ausgewertet und in pseudonymisierter Form gespeichert werden. Ich bin mir darüber bewusst, dass meine Teilnahme freiwillig erfolgt und ich die Teilnahme jederzeit und ohne die Angabe von Gründen abbrechen kann.

______________	______________________________	____________________________
Datum	Name (in Druckschrift)	Unterschrift (Patient*in)
______________	______________________________	____________________________
Datum	Name (in Druckschrift)	Unterschrift (Studienverantwortliche*r)

Bei Fragen, Anregungen oder **Beschwerden** können Sie sich gerne an den Projektleiter wenden:
Prof. Dr. med. Georgios Paslakis, Leitender Arzt, Universitätsklinik für Psychosomatische Medizin und Psychotherapie, Ruhr Universität Bochum – Campus OWL, Virchowstr. 65, 32312 Lübbecke
Telefon: 05741-35-474500; Email: Georgios.Paslakis@ruhr-uni-bochum.de

Verantwortliche Person für die Datenverarbeitung dieser Studie:
Prof. Dr. med. Georgios Paslakis, Leitender Arzt, Universitätsklinik für Psychosomatische Medizin und Psychotherapie, Ruhr Universität Bochum – Campus OWL, Virchowstr. 65, 32312 Lübbecke
Telefon: 05741-35-474500; Email: Georgios.Paslakis@ruhr-uni-bochum.de

Bei Fragen zum Datenschutz kann auch der Datenschutzbeauftragte der Universitätsklinik für Psychosomatische Medizin und Psychotherapie in Lübbecke kontaktiert werden:
[personal information redacted]
Oder auch die Landesbeauftragte NRW:
[personal information redacted]

English Translation of Consent Form

Reducing the urge to be physically active in anorexia nervosa using virtual reality:
A randomized controlled feasibility study

Patient information and informed consent

Patient information

The guidelines of good clinical practice (GCP) stipulate that participants in empirical studies must explicitly and comprehensibly declare that they are voluntarily participating in our research. For this reason, we would like to ask you to read the following explanations on the content of the study and to indicate whether you agree to participate in the study and consent to the processing of your data.

Investigator and trial site of this study

Prof. Dr. med. Georgios Paslakis, MBA
University Clinic for Psychosomatic Medicine and Psychotherapy
Virchowstraße 65 - 32312 Lübbecke
Telephone: 05741 / 35 - 4500 (secretary's office) - Fax: 05741 / 35 – 2710
E-mail address: psychosomatik@muehlenkreiskliniken.de

Subject of the study

People with anorexia nervosa ("anorexia nervosa") often feel the urge to exercise excessively or to be physically active in other ways, which can have a negative impact on their health. However, the acute urge to be physically active is rarely taken into account in the treatment of eating disorders. The aim of the present study, "Reducing the urge to be physically active in anorexia nervosa using virtual reality: a randomized controlled feasibility study", is to test a virtual reality (VR)-based approach to alleviate the urge for excessive activity. Virtual reality (VR) describes an artificial, computer-generated environment that is displayed using special glasses, in which you can appear to move and perform actions.

Procedure of the study

This study involves answering questions about yourself, questionnaires on eating disorder symptoms and the urge to be physically active, as well as repeated participation in and evaluation of an exercise in "virtual reality", which is displayed using glasses.

Duration

Participation in the study lasts a total of 4 weeks. We will ask you to fill out a series of questionnaires to include you in the study. This will take about 45 minutes. We will then arrange two sessions of 30 minutes each per week for a total of 4 weeks. The sessions will include 20 minutes each where you will wear the VR goggles and complete an exercise while seated, and 10 minutes where you will answer questions about the exercise and/or further questionnaires about the urge to be physically active and eating disorder symptoms.

Possible benefits of the study

The intervention of this study aims to reduce a perceived urge to be physically active. However, you may not derive any direct health or personal benefit from your participation, but future patients with eating disorders will benefit from the results of this study.

Experiences/risks associated with participation

Participants in this study will not be exposed to any health risk beyond the risks of everyday life. Some people experience nausea when they see movement in virtual reality without moving themselves. You are free to end your participation at any time.

Data protection declaration

The data processing of this study is carried out in accordance with the data protection provisions of the General Data Protection Regulation (GDPR) and the Data Protection Act of North Rhine-Westphalia (DSG NRW). The data will be used exclusively for the purposes described in the information sheet.

The following data is collected as part of this study:

- Questionnaire to record eating disorder symptoms (EDE-Q)
- Questionnaire to record the urge to be physically active (SUPA-Q)
- Questionnaire to record simulation sickness (SSQ)
- Questionnaire for the assessment of simulation usability (SUS)
- Questionnaire for the assessment of psychotherapy motivation (FPTM-23)
- Questionnaire to record driven sporting activity (CES)

Personal data that is collected:

- Age
- Gender
- German language skills
- educational status
- Height
- Body weight (from the patient file)
- Medication
- Time since first diagnosis of an eating disorder
- Number of previous treatments
- Diagnosis (from the patient file)

Confidentiality

All data collected as part of this study is of course confidential and will only be used in pseudonymized form. Demographic data such as age or gender do not allow any clear conclusions to be drawn about your person.

Note on the use of products from Meta Platforms, Inc.

Products from Meta Platforms, Inc. are used in this study (VR glasses and control units, software for graphical representation). The use of these products does not require the collection of your personal data and this data is not collected or stored within the hardware and software. Meta Platforms, Inc. does not receive any access to the data collected here. However, by participating, you agree to the Meta Platforms, Inc. terms of use and acknowledge that the use of the products does not give rise to any guarantee, warranty or liability claims against Meta Platforms, Inc.

Storage

The data collected in this study will be stored at the site of the University Clinic for Psychosomatic Medicine and Psychotherapy in Lübbecke and deleted after 10 years. The data is stored in a form that does not allow any direct conclusions to be drawn about your person, i.e. the data is pseudo-nymized.

Voluntariness & rights of the participants

Your participation in this study is voluntary. Until the results of the study are published, you are free to discontinue your participation and thus withdraw your consent (revocation) without any disadvantages for you. If you withdraw your participation, we will ask you whether we may use the data collected up to the time of withdrawal; if you do not agree to this, all available data relating to you will be destroyed.

You have the right to obtain information about the personal data concerning you and, if necessary, to request that it be corrected or deleted. In the event of a dispute, you have the right to lodge a complaint with the data protection officer of the University Clinic for Psychosomatic Medicine and Psychotherapy Lübbecke or with the data protection officers of the state of North Rhine-Westphalia (see declaration of consent for address).

Declaration of consent

I have read the explanations and agree to participate in the above-mentioned study. I have had the opportunity to ask questions and clarify any ambiguities. I have received a copy of this declaration.

I agree that the data collected as part of the study may be evaluated for scientific purposes and stored in pseudonymized form. I am aware that my participation is voluntary and that I can withdraw at any time without giving reasons.
